# Natural Host Genetic Resistance to Lentiviral CNS Disease: A Neuroprotective MHC Class I Allele in SIV-Infected Macaques

**DOI:** 10.1371/journal.pone.0003603

**Published:** 2008-11-03

**Authors:** Joseph L. Mankowski, Suzanne E. Queen, Caroline S. Fernandez, Patrick M. Tarwater, Jami M. Karper, Robert J. Adams, Stephen J. Kent

**Affiliations:** 1 Department of Molecular and Comparative Pathobiology, Johns Hopkins University School of Medicine, Baltimore, Maryland, United States of America; 2 Department of Pathology, Johns Hopkins University School of Medicine, Baltimore, Maryland, United States of America; 3 Department of Neurology, Johns Hopkins University School of Medicine, Baltimore, Maryland, United States of America; 4 Department of Microbiology and Immunology, University of Melbourne, Parkville, Victoria, Australia; 5 Department of Biomedical Sciences, Texas Tech University Health Sciences Center, Paul L. Foster School of Medicine, El Paso, Texas, United States of America; Institut Pasteur Korea, Republic of Korea

## Abstract

Human immunodeficiency virus (HIV) infection frequently causes neurologic disease even with anti-retroviral treatment. Although associations between MHC class I alleles and acquired immunodeficiency syndrome (AIDS) have been reported, the role MHC class I alleles play in restricting development of HIV-induced organ-specific diseases, including neurologic disease, has not been characterized. This study examined the relationship between expression of the MHC class I allele Mane-A*10 and development of lentiviral-induced central nervous system (CNS) disease using a well-characterized simian immunodeficiency (SIV)/pigtailed macaque model. The risk of developing CNS disease (SIV encephalitis) was 2.5 times higher for animals that did not express the MHC class I allele Mane-A*10 (*P* = 0.002; RR = 2.5). Animals expressing the Mane-A*10 allele had significantly lower amounts of activated macrophages, SIV RNA, and neuronal dysfunction in the CNS than Mane-A*10 negative animals (*P*<0.001). Mane-A*10 positive animals with the highest CNS viral burdens contained SIV *gag* escape mutants at the Mane-A*10-restricted KP9 epitope in the CNS whereas wild type KP9 sequences dominated in the brain of Mane-A*10 negative animals with comparable CNS viral burdens. These concordant findings demonstrate that particular MHC class I alleles play major neuroprotective roles in lentiviral-induced CNS disease.

## Introduction

With 33 million people infected with HIV (UNAIDS 2007), unraveling the pathogenesis of this infection is critical. In addition to immunosuppression manifest as AIDS, HIV infection frequently causes neurologic disease ranging from subtle cognitive deficits to overt dementia, often occurring despite anti-retroviral treatment [1], [2]. The neuropathogenesis of HIV infection remains incompletely understood.

MHC class I-restricted CD8^+^ T cell responses are a critical part of the adaptive cell-mediated immune response to HIV-1 infection of humans and SIV infection of macaques [3], [4]. The HLA-B*27 and HLA-B*5701 MHC class I alleles have been associated with slower progression to AIDS with maintenance of CD4^+^ T cell counts [5]–[7]. Similarly, the presence of the MHC class I alleles Mane-A*10 in pigtailed macaques and Mamu-A*01 in rhesus macaques have been linked to slower progression to AIDS following SIV infection [8]–[11]. Despite these provocative relationships between MHC class I alleles and development of the syndrome AIDS, associations between MHC class I alleles and HIV-induced organ-specific disease outcomes including HIV-associated neurocognitive disorders have not been identified. CD8^+^ T cells are present in high numbers in the brain of HIV-infected patients during asymptomatic infection, supporting the concept that effective cytotoxic T cell control of HIV/SIV in the CNS may be crucial to prevent lentiviral CNS disease [12], [13].

To facilitate pathogenesis studies, we have established an accelerated SIV/macaque model of HIV-induced CNS disease. In this model, pigtailed macaques (*Macaca nemestrina*) are inoculated simultaneously with a cloned neurovirulent virus, SIV/17E-Fr, and an immunosuppressive swarm, SIV/DeltaB670. Using this dual infection protocol, the majority of infected animals develop prototypic SIV encephalitis by three months post-infection that closely resembles HIV encephalitis [14].

The provocative finding that some SIV-infected pigtailed macaques do not develop CNS disease (although all animals develop similar high levels of persistent viremia and progress to AIDS) suggests that there are host genetic factors that confer resistance to lentiviral-induced CNS disease. To determine whether MHC class I allele expression patterns could explain the variable progression to SIV-induced CNS disease, we established which MHC class I alleles were expressed by 63 pigtailed macaques and then compared MHC class I allele expression with CNS disease outcome following SIV infection.

## Materials and Methods

### Animals

This retrospective cohort study included 63 pig-tailed macaques (*Macaca nemestrina*) that were intravenously inoculated with SIV/DeltaB670 (50 AID_50_), and SIV/17E-Fr (10,000 AID_50_). Treatment of SIV-infected pigtailed macaques included: IFNβ (1.3 ug/kg every 3 days; n = 6), minocycline (2 mg/kg twice daily; n = 14), 9-[(R)-2-phosphonylmethoxy) propyl] adenine (PMPA; 30mg/kg once daily; n = 5), or PMPA with minocycline (n = 6)[15]. Animals were perfused with sterile saline at euthanasia to remove blood and circulating virus from brain. The animal procedures in this study were performed according to the principles set forth by the Institutional Animal Care and Use Committee at Johns Hopkins University and the National Research Council's Guide for the care and use of laboratory animals.

### Immunohistochemical staining and histopathology

Primary antibody against CD68 (KP-1, diluted 1∶2,000, DAKO, Carpinteria, CA), a marker of microglial activation and macrophage infiltration, was used to immunostain brain sections. To identify β-APP accumulation in axons, coronal sections of brain tissue including basal ganglia, frontal cortex, and corpus callosum, were immunohistochemically stained with the monoclonal antibody anti-β-amyloid precursor protein 695 (Clone LN27, Zymed, South San Francisco, CA). All brain tissue sections were stained by an automated immunostainer (Optimax Plus, BioGenex, San Ramon, CA) for uniformity. Streck-fixed, paraffin-embedded brain tissue sections were deparaffinized, rehydrated, and then post-fixed in Streck tissue fixative (Streck Laboratories, Omaha, NE) for 20 minutes. After rinsing in water, tissues were heated in a microwave in sodium citrate buffer (0.01M, pH 6.0) for 8 minutes to retrieve antigen. Endogenous peroxidase was quenched with 3% H_2_O_2_ for 10 minutes and then sections were blocked with buffered casein for 10 minutes. Primary antibody was applied to tissue sections for 60 minutes at room temperature, the tissues were washed in buffer, and then secondary biotinylated multilink antibody (Biogenex, San Ramon, CA) was added for 20 minutes. After washing, streptavidin-horseradish peroxidase was applied for 20 minutes, followed by diaminobenzidene tetrahydrochloride in buffer containing H_2_O_2_ for 10 minutes. Sections were then washed, dehydrated and mounted [16], [17].

### Quantitative image analysis

**Figure 1:**
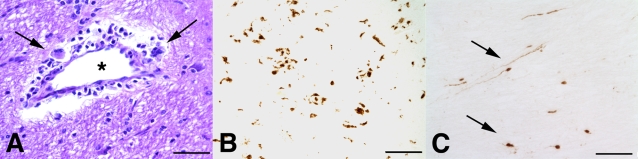
SIV infection induced encephalitis, increased CNS CD68 expression, and axonal accumulation of APP. SIV-infected pig-tailed macaques that developed encephalitis had multifocal perivascular accumulations of infiltrating macrophages and multinucleate giant cells (A, arrows denote giant cells, * denotes blood vessel lumen, hematoxylin and eosin stain, bar = 50 uM), increased CNS CD68 expression reflecting activation of macrophages and microglia in subcortical white matter shown by immunohistochemical staining with an anti-CD68 antibody (B, bar = 50 uM), and accumulation of APP in axons (arrows) in the corpus callosum (C, bar = 100 uM).

To standardize sampling from animal to animal, coronal brain tissue sections from all animals were prepared from the same location in the basal ganglia, 5 mm posterior to the head of the caudate nucleus. For each animal, twenty adjacent fields in the corpus callosum from sections immunostained for APP were captured at 200× magnification (an area of 2.8 mm^2^) using a Sensys 2 digital camera (Photometrics, Tucson, AZ) then analyzed by IP Lab imaging software (Scanalytics, Vienna, VA). Image analysis transects of the corpus callosum started on the midline and proceeded laterally, encompassing the identical region of corpus callosum in all animals. Similarly, twenty 200× fields were captured in subcortical white matter subjacent to cingulate gyrus for measurement of CD68 representing macrophage/microglial activation (**Figure 1**). Images were binarized and the total area occupied by immunopositive pixels calculated to measure the total area of immunostaining [16], [17].

### Measuring SIV RNA by real-time RT-PCR

Viral RNA was quantitated in the basal ganglia of brain by real-time PCR using primers in the SIV *gag* region on an Applied Biosystems Prism 5700 Sequence Detection System. The primers to detect unspliced viral RNA included (SGAG03–5′- CAGGGA AII AAG CAG ATG AAT TAg–3′: SGAG04–5′- GTTTCACTTTCTCTTCTGCGT g–3′ and pSUS05–5′ (FAM)ATTTGGATTAGCAGAAAGCCTGTTGGAG (TAMRA+BLOCKED)–3′) [18].

### Reference-strand mediated conformational analysis

Pigtail macaque class I sequences spanning 200 bp of the polymorphic peptide binding regions were amplified using Phusion DNA polymerase (Finnzymes, Espoo, Finland) using 1 µl of a 25 µM phosphate-labelled forward primer (5′Phos-shtRSCA; 5′-[phos]-AggggCCggAgTATTggg-3′) and an unlabelled reverse primer (3′sht-RCSA; 5′-TTCAggRCgAWgTAATCC-3′), for an initial 30 s 98°C step followed by 35 cycles of 98°C 5 s, 55°C 1s, and 72°C 20 s, then a final extension at 72°C 5 minutes. Reference strands were amplified under the same conditions, but with a FAM-labeled forward primer (5′FAM-shtRSCA; 5′[6-FAM]-AggggCCggAgTATTggg-3′) and a phosphate-labelled reverse primer (3′Phos-shtRSCA; 5′-[phos]-TTCAggRCgAWgTAATCC-3′). Following amplification, samples were digested for 30 minutes at 37°C with 10 U lambda exonuclease (NE Biolabs, Ipswich, MA, USA). The single-stranded cDNA amplicons were then heteroduplexed with the fluorescently labelled reference strand in a reaction involving 4 minutes at 95°C, 5 minutes at 55°C, and 15 minutes at 15°C. The labelled heteroduplexes were run on a non-denaturing gel (LongRanger gel mix, Cambrex, Mt. Waverly, VIC, Australia) on an ABI PRISM 377 DNA Sequencer (Applied Biosystems, Foster City, CA, USA), where each heteroduplex displays a characteristic mobility. The pattern of heteroduplexes from each reaction were then compared to the mobilities of previously characterized, sequence-verified *Mane* MHC class I clones using DAx data acquisition and analysis software (Van Mierlo Software, Eindhoven, the Netherlands) [9], [19].

### Sequence-specific PCR for Mane-A*10 expression

RNA was isolated from pre-inoculation PBMCs and cDNA was generated. Initial PCR was performed for Sequence Specific Primer (SSP) 2 (forward 5′-CGG GTC TCA CAC CTT CCA GAG GAT GTA T-3′ reverse 5′-CGG TCC AGG AAC GCA GGT CCC-3′) and GAPDH (forward 5′-TGC CAT CAA TGA CCC CTT CAT TGA CCT C-3′ reverse 5′-CCC AGC CTT CTC CAT GGT GGT GAA GAC-3′) under the following cycling conditions: 95°C 20 min followed by 35 cycles 94°C 20 sec, 72°C 40 sec final extension 72°C 10 min. Any samples with a 134 bp amplified product were subsequently run for SSP1 (forward 5′-GGC CAA CAC ACA GAC CTA CCG AGA GAG-3′ reverse 5′-CCC TGC CGT CGT AGG CGT ACT GGC TAT AT-3′ 161 bp) and SSP3 (forward 5′-GGC GCC TCC TCC GCG GAT ATA G-3′ reverse 5′-GGC ACT CGC CCT CCA CGT AGG T-3′ 174 bp) in addition to SSP2 and GAPDH repeat. Samples which were negative for SSP2 were repeated to verify their Mane-A*10 status while samples positive for SSP2, SSP1, and SSP3 were considered Mane-A*10 positive.

### Cloning and sequencing of the SIV KP9 gag epitope

PCR was performed on cDNA prepared from inoculum viral stock RNA or RNA extracted from the basal ganglia using the SIV *gag* KP9-specific primers forward 5′-CAC GCA GAA GAG AAA GTG AA-3′ and reverse 5′-GTT CCT CGA AT(AG) TC(GT) GAT CC-3′ using Platinum PCR supermix (Invitrogen, Carlsbad, CA) and the following cycle conditions: 94°C for 2 min., 30 cycles at 94°C for 15 sec, 56°C for 30 sec, and 72°C for 1 min., followed by a final extension of 72°C for 8 min [20]. The bulk PCR product was then cloned into pCRII vector using the TOPO TA cloning kit (Invitrogen). Colonies were plated on LB Kanamycin with X-gal and grown overnight. Colonies were selected and grown in LB Kan 10% glycerol for 12 h statically and sequenced by Agencourt Biosciences (Beverly, MA). Sequences were aligned and analyzed using Geneious 3.0.3 software.

### Statistical Methods

**Figure 2:**
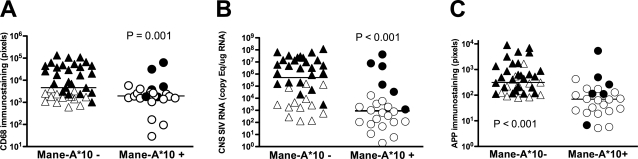
Reduced CNS macrophage activation, SIV replication, and axonal APP accumulation associated with Mane-A*10 expression. Mane-A*10 expression was associated with reduced CNS inflammation, SIV replication and neuronal damage in SIV-infected macaques. A) SIV-infected macaques expressing the Mane-A*10 allele (circles) had significantly lower CNS macrophage infiltration and activation than animals without the allele (triangles, *P* = 0.001) shown by measuring the amount of CD68 immunostaining in subcortical white matter of the brain. B) Animals expressing the Mane-A*10 allele (circles) also had significantly lower SIV RNA in the basal ganglia of the brain than animals without the allele (triangles, *P*<0.001). SIV RNA was measured by real time RT-PCR. C) Animals expressing the Mane-A*10 allele (circles) also had significantly lower axonal accumulation of APP in the corpus callosum than animals without the allele (triangles, *P*<0.001,) demonstrating that expression of Mane-A*10 is neuroprotective. Solid (black) symbols represent the SIV-infected animals that developed SIV encephalitis whereas the open symbols indicate animals that were SIV-infected but did not develop SIV encephalitis.

**Figure 3:**
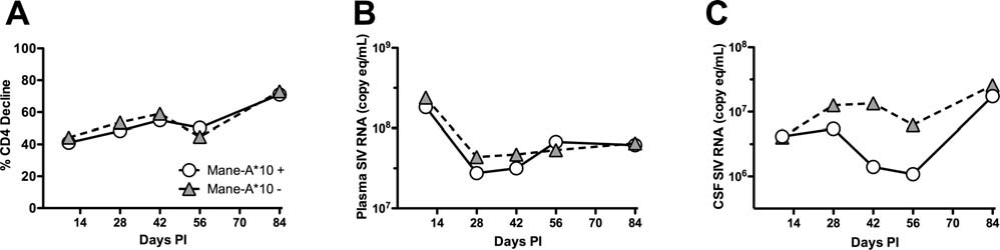
Mane-A*10 expression did not influence CD4+ T cell decline or plasma viral load but was associated with lower CSF viral loads. Comparing longitudinal trends in CD4+ T cell decline from baseline pre-infection values and plasma SIV RNA demonstrated that Mane-A*10 expression was not associated with either extent of CD4+ T cell decline throughout infection (A) or with altered plasma viral load set points in untreated SIV-infected macaques at any time point from primary through asymptomatic to terminal stages of (B). To determine whether Mane-A*10 expression was associated with altered viral replication in the periphery, mean plasma viral load throughout infection was measured in untreated SIV-infected animals grouped by Mane-A*10 status (Mane-A*10 positive animals represented by circles, n = 11; Mane-A*10 negative animals represented by triangles, n = 21). Plasma viral load also did not differ significantly between these groups at any time-point from day 14 post-inoculation until terminal sampling (*P*>0.05). Similarly, Mane-A*10 expression status was not associated with extent of % CD4+ T cell decline in SIV-infected macaques, with no statistically significant difference between groups of animals composed of Mane-A*10 positive animals (circles) versus Mane-A*10 negative macaques (triangles). Combined, these data indicate that neither plasma viral load or CD4+ T cell loss are associated with expression of Mane-A*10 in pigtailed macaques inoculated with SIV/17E-Fr and SIV/DeltaB670. In contrast, mean SIV RNA levels in CSF were lower in the group of Mane-A*10 positive animals (circles) versus Mane-A*10 negative macaques (triangles).

Comparisons of biomarkers (e.g., CD68, APP, CNS SIV RNA level) between groups (Mane-A*10 allele present versus Mane-A*10 allele absent) utilized Student's two sample t-test. A log 10 transformation of CD68, APP, and CNS SIV RNA measurements was performed before the use of the t-test to ensure the data were more normally distributed than in original scale (**Figure 2**). In addition, t-tests were performed at each day post-inoculation to identify differences, if any, between the Mane-A*10 groups for those biomarkers measured over time (**Figure 3**). Risk ratios and Fisher's exact test were conducted to identify the magnitude and significance, if any, of the association of Mane-A*10 allele expression and SIV CNS disease (**Table 1**). In addition, to evaluate the possible influence of treatment on this association, the Mantel-Haenzel homogeneity of risk ratios test was conducted across the treatment strata-specific risk ratios. The null assumption of this test is that treatment does not modify the effect measure of the Mane-A*10 and SIV CNS disease association whereby one could then combine the frequencies across the strata (when *P*>0.05). Risk ratios are presented for this study because the design was a retrospective (historic) cohort study, which means the animals were included in this study based on their exposure status (Mane-A*10), as opposed to their disease status (SIV CNS disease), and followed per study protocol to determination of disease status.

**Table 1:** Mane-A*10 Status versus Development of SIV Encephalitis.

	Untreated		Treated	
Mane-A*10 allele	SIV CNS Disease	No SIV CNS Disease	SIV CNS Disease	No SIV CNS Disease
Absent	17	4	9	9
Present	4	7	2	11
Strata Specific Risk Ratio (*P*-value)	2.2 (0.020)		3.3 (0.066)	
Combined Risk Ratio (*P*-value)	2.5 (0.002)*			

The Mantel-Haenzel test of homogeneity (^*^) failed to reject the hypothesis that the strata specific risk ratios were equal (*P* = 0.632).

## Results and Discussion

In this study, a well-characterized SIV/pigtailed macaque model of HIV CNS disease was examined to determine whether expression of the MHC class I allele Mane-A*10 influenced SIV CNS disease outcome [14], [16], [17], [21]. 24 of 63 (38%) pigtailed macaques included in this study expressed the Mane-A*10 allele as determined by reference strand-mediated conformational analysis and/or sequence-specific PCR performed as reported previously [19]. SIV encephalitis, with multifocal perivascular and parenchymal accumulations of SIV-infected macrophages and multinucleate giant cells (**Figure 1A**), was identified in 32 of 63 macaques infected with both SIV/17E-Fr and SIV Delta/B670 by examining brain tissue sections microscopically [16]. SIV-infected macaques with encephalitis typically have concordant increases in CNS immunostaining for the macrophage activation marker CD68 (**Figure 1B**) and increases in accumulation of amyloid precursor protein (APP) in axons reflecting neuronal dysfunction (**Figure 1C**).

Only six of 24 (25%) SIV-infected pigtailed macaques expressing the Mane-A*10 allele developed SIV CNS disease whereas 26 of 39 (67%) SIV-infected animals lacking the Mane-A*10 allele developed SIV CNS disease (**Table 1**). Therefore, macaques not expressing the Mane-A*10 allele were 2.5 times as likely to develop SIV CNS disease than macaques expressing the Mane-A*10 allele, demonstrating that expression of Mane-A*10 is neuroprotective in SIV-infected pigtailed macaques (*P* = 0.002, Fisher's exact test; risk ratio RR = 2.5). Mane-A*10 positive animals also had lower amounts of CNS immunostaining for CD68 (reflecting decreased SIV-induced activation of both infiltrating macrophages and resident microglia in the brain) than Mane-A*10 negative animals (**Figure 2A**; *P* = 0.001) consistent with the reduced incidence of SIV encephalitis in animals with the Mane-A*10 allele. The presence of macrophages and microglia in the brain of HIV-infected individuals has been highly correlated with the severity of HIV-induced dementia [22].

We subsequently examined whether SIV-infected pigtailed macaques expressing the Mane-A*10 allele had altered levels of viral replication in the CNS versus animals without the allele by measuring SIV RNA in the basal ganglia via real time RT-PCR. Macaques with the Mane-A*10 allele had significantly lower levels of SIV RNA in the basal ganglia of the brain versus animals without the Mane-A*10 allele (**Figure 2B**; *P*<0.001).

We then measured accumulation of amyloid precursor protein (APP) in the corpus callosum of SIV-infected macaques as a marker of neuronal dysfunction to verify that expression of the Mane-A*10 allele also conferred protection against neuronal damage [16]. Animals with the Mane-A*10 allele had significantly lower amounts of APP (measured by immunostaining and digital image analysis) than animals that did not express the allele (**Figure 2C**, *P*<0.001). Although we did not measure behavioral deficits in all of the SIV-infected animals in this study, we previously have demonstrated that the amount of both APP accumulation and CD68 immunostaining are strongly correlated with impaired fine-motor control in this SIV/macaque model, suggesting that animals expressing the Mane-A*10 allele also may be protected against behavioral impairment caused by SIV [23].

The concordant findings of decreased incidence of SIV encephalitis, lower macrophage activation, markedly reduced CNS viral load, and decreased APP accumulation in axons in pigtailed macaques positive for the Mane-A*10 allele strongly suggest that particular MHC class I alleles, such as Mane-A*10, play major neuroprotective roles in lentiviral-induced CNS disease. As the magnitude of per cent decline in circulating CD4^+^ T cell counts from pre-inoculation values to terminal time-points was not associated with Mane-A*10 status (**Figure 3A**), the protective effects of Mane-A*10 conferring resistance to SIV-induced CNS disease appear to be independent of its influence on AIDS progression in this SIV/macaque model. Furthermore, in this study, pigtailed macaques expressing Mane-A*10 inoculated simultaneously with both SIV/DeltaB670 and SIV/17E-Fr did not have significantly lower plasma viral loads at any time point post-infection than animals not expressing Mane-A*10 (**Figure 3B**), even though CNS SIV RNA levels were markedly lower in Mane-A*10 positive animals. Combined, these findings demonstrate that the expression of Mane-A*10 in this SIV model is specifically associated with improved CNS disease outcome and is not simply a reflection of slower progression to AIDS or lowered viral replication in the periphery. This is consistent with our previous finding that plasma viral load does not predict SIV-induced CNS disease in this SIV/macaque model [17]. In contrast with plasma viral load, CSF viral load was lower in SIV-infected macaques expressing the Mane-A*10 allele (**Figure 3C**) reflecting lowered CNS viral replication after day 14 post-inoculation in animals expressing Mane-A*10. The difference in CSF vRNA was significantly lower at day 42 in the group expressing Mane-A*10 (*P* = 0.029) versus the group without Mane-A*10. It is likely that the groups were not significantly different at later time points because 1) viral escape developed in some of the Mane-A*10 positive animals and 2) the CSF does not solely represent CNS viral replication but also reflects SIV replication in other compartments including meninges and blood.

The lack of a reduction in peripheral (i.e. plasma) viral load in the Mane-A*10 positive pigtailed macaques inoculated intravenously with SIV/DeltaB670 and SIV/17E-Fr in this report is in distinction to our previous work that evaluated 8 animals (of which 3 were Mane-A*10 positive) infected with SIV/mac251 intrarectally. Several factors likely account for this difference including different viruses that were used for inoculation, inoculation route, inoculation dose, and host genetic factors in addition to Mane-A*10. Furthermore, peripheral immune escape in some animals may play a role in abrogating any Mane-A*10-related affect on peripheral viral load. We previously showed that Mane-A*10 positive animals infected with SIV/mac251 that undergo immune escape at KP9 have viral loads that resemble those of Mane-A*10 negative macaques [9].

32 of the 63 SIV-infected animals examined in this study did not receive treatment during infection; the other 31 SIV-infected pigtailed macaques received IFNβ, minocycline, 9-[(R)-2-phosphonylmethoxy) propyl] adenine (PMPA), or PMPA with minocycline (as detailed in Materials and Methods). To examine the possibility that the Mane-A*10 and SIV CNS disease association was modified by treatment during SIV infection, the relative risks were calculated separately for SIV-infected macaques that either did or did not receive treatment (**Table 1**). The strata-specific risk ratio for the group of untreated SIV-infected macaques was 2.2 (*P* = 0.02, Fisher's exact) and the relative risk for the treated group of SIV-infected macaques was 3.3 (*P* = 0.066, Fisher's exact). A significant difference in risk of SIV CNS disease versus expression of Mane-A*10 was not found between treated and untreated groups of SIV-infected macaques indicating that the Mane-A*10 expression was neuroprotective even in treated SIV-infected animals (Mantel-Haenzel test of homogeneity; *P* = 0.632). Thus, it is likely that MHC class I alleles exert a similar neuroprotective effect in HIV-infected individuals irrespective of treatment status.

In pigtailed macaques, Mane-A*10 allele restriction of the immunodominant SIV Gag epitope KP9 has been associated with lowered plasma viral loads after intra-rectal SIVmac251 inoculation [8], [9]. Conversely, escape mutations in the SIV KP9 Gag epitope are present at high frequency in Mane-A*10 positive animals with high plasma viral loads. In this study, to determine whether viral escape at the immunodominant SIV Gag KP9 epitope could be detected in the CNS of animals with the Mane-A*10 allele that developed SIV CNS disease with high levels of vRNA in brain, cDNA samples prepared from brain RNA from the six Mane-A*10 positive animals with SIV encephalitis were bulk PCR amplified and sequenced. SIV *gag* sequences also were determined for six Mane-A*10 negative macaques with comparable levels of SIV RNA in the CNS and for the viral stocks of SIV/DeltaB670 and SIV/17E-Fr used to inoculate macaques (**Figure 2B** and **Table 2**). The four Mane-A*10 positive animals (animals 18292, PVg2, A4P012, and A2P005) with the highest CNS viral burdens contained *gag* escape mutants (K165R) in the CNS. In contrast, wild type KP9 sequences strongly dominated in the brain of Mane-A*10 negative animals (**Table 2**). This difference illustrates that viral escape mutations are present in the CNS and may be playing a pivotal role in the development of lentiviral-induced encephalitis.

**Table 2:** Viral Escape in the CNS of Mane-A*10 Animals with SIV Encephalitis.

Inoculum		Gag 164-172	Frequency	
SIV/17E-Fr		KKFGAEVVP	100%	
SIV/DeltaB670		---------	100%	
Mane-A*10 ** positive**	**CNS SIV RNA (Copy eq/ug RNA**)			
18292	4.2×10^7^	-R-------	100%	(10/10)
PVg2	9.9×10^6^	---------	33%	(16/48)
PVg2		-R-------	67%	(32/48)
A4P012	4.6×10^6^	---------	90%	(9/10)
A4P012		-R-------	10%	(1/10)
A2P005	1.4×10^5^	-R-------	100%	(12/12)
T4363	3.5×10^4^	---------	100%	(42/42)
DM19	1.2×10^3^	---------	100%	(11/11)
Mane-A*10 ** negative**				
A4P028	1.2×10^8^	---------	100%	-
PNw1	6.8×10^7^	---------	100%	-
A1P014	3.4×10^7^	---------	100%	-
A1P005	2.9×10^7^	---------	100%	(11/11)
CC33	1.1×10^6^	---------	100%	(12/12)
BM03	2.0×10^5^	---------	100%	(12/12)

The four Mane-A^*^10 positive animals (animals 18292, PVg2, A4P012, and A2P005) with the highest CNS viral burdens contained high levels of *gag* escape mutants (K165R) in the CNS. In contrast, wild type KP9 sequences strongly dominated in the brain of Mane-A^*^10 negative animals. Wild type KP9 sequences also were exclusively detected in viral stocks of SIV/DeltaB670 and SIV/17E-Fr used for inoculation.

Future studies to evaluate the longitudinal evolution of escape mutants in the CNS versus plasma would determine whether selection pressure on SIV Gag varies between these distinct compartments and whether Gag escape mutants could arise in the CNS independently from the periphery and then traffic from the brain to seed the periphery. Our previous studies comparing SIV genotypes in the CNS versus plasma have suggested that latent SIV can emerge from the CNS, traffic out of the brain, and then replicate in the periphery [24].

SIV/macaque models are widely used to define the pathogenesis of HIV infection and for development of effective preventive strategies. Identifying the MHC class I alleles that influence SIV disease progression is crucial for refining our understanding of SIV/macaque models especially because macaque studies frequently employ relatively small numbers of animals. As this study illustrates, the impact of MHC class I alleles on SIV disease progression can extend beyond influencing the time course of AIDS onset or plasma viral load set point. In fact, MHC class I allele effects may be strongest in modifying organ-specific diseases induced by HIV independent of any appreciable effects in the periphery. The distinct neuroprotective influence of Mane-A*10 on SIV-induced CNS disease also demonstrates that the relative efficacy of cell-mediated control of pathogens in the CNS can be a distinct process from cell-mediated immune control in the periphery. Some SIV/macaque models used to study HIV CNS disease have relied on antibody-mediated depletion of CD8+ T cells to increase the incidence of CNS disease. This strategy may compromise pathogenesis studies in the SIV/macaque model because 1) cell-mediated immune responses are eliminated that play crucial roles in controlling viral replication in the CNS of HIV-infected individuals and 2) CD8+ T cell depletion decreases immune mediated selection pressure on viral evolution in the CNS. Additional, as yet unidentified, MHC I and II alleles and polymorphisms within genes across extended MHC haplotypes are also likely to influence CNS disease and other organ-specific diseases[25].

This study shows that MHC class I-restricted CTL responses against SIV Gag play a major role in preventing lentiviral-induced CNS disease. Strategies aimed at stimulating MHC class I-restricted CD8 T cell responses against immunodominant HIV epitopes may be of great value to prevent as well as treat HIV CNS disease. The identification of a neuroprotective MHC class I allele in this SIV/macaque model sets the stage for performing parallel studies in neuroAIDS cohorts to determine whether particular MHC class I alleles may protect HIV-infected individuals from HIV-associated neurologic disorders.
